# Critical inspiratory pressure – a new methodology for evaluating and training the inspiratory musculature for recreational cyclists: study protocol for a randomized controlled trial

**DOI:** 10.1186/s13063-019-3353-0

**Published:** 2019-05-07

**Authors:** Patricia Rehder-Santos, Vinicius Minatel, Juliana Cristina Milan-Mattos, Étore De Favari Signini, Raphael Martins de Abreu, Carla Cristina Dato, Aparecida Maria Catai

**Affiliations:** 1 Cardiovascular Physical Therapy Laboratory, Nucleus of Research in Physical Exercise, Department of Physical Therapy, Federal University of São Carlos, Via Washington Luiz, km 235, CP: 676, São Carlos, São Paulo 13565-905 Brazil; 2 Nutrition Course, Central University of Paulista, São Carlos, São Paulo Brazil

**Keywords:** Physical exercise, Physiotherapy, Physical performance, Critical power, Respiratory muscle

## Abstract

**Background:**

Inspiratory muscle training (IMT) has brought great benefits in terms of improving physical performance in healthy individuals. However, there is no consensus regarding the best training load, as in most cases the maximal inspiratory pressure (MIP) is used, mainly the intensity of 60% of MIP. Therefore, prescribing an IMT protocol that takes into account inspiratory muscle strength and endurance may bring additional benefits to the commonly used protocols, since respiratory muscles differ from other muscles because of their greater muscular resistance. Thus, IMT using critical inspiratory pressure (PThC) can be an alternative, as the calculation of PThC considers these characteristics. Therefore, the aim of this study is to propose a new IMT protocol to determine the best training load for recreational cyclists.

**Methods:**

Thirty recreational cyclists (between 20 and 40 years old) will be randomized into three groups: sham (SG), PThC (CPG) and 60% of MIP, according to age and aerobic functional capacity. All participants will undergo the following evaluations: pulmonary function test (PFT), respiratory muscle strength test (RMS), cardiopulmonary exercise test (CPET), incremental inspiratory muscle endurance test (iIME) (maximal sustained respiratory pressure for 1 min (PTh_MAX_)) and constant load test (CLT) (95%, 100% and 105% of PTh_MÁX_) using a linear load inspiratory resistor (PowerBreathe K5). The PThC will be calculated from the inspiratory muscle endurance time (T_LIM_) and inspiratory loads of each CLT. The IMT will last 11 weeks (3 times/week and 55 min/session). The session will consist of 5-min warm-up (50% of the training load) and three sets of 15-min breaths (100% of the training load), with a 1-min interval between them. RMS, iIME, CLT and CPET will be performed beforehand, at week 5 and 9 (to adjust the training load) and after training. PFT will be performed before and after training. The data will be analyzed using specific statistical tests (parametric or non-parametric) according to the data distribution and their respective variances. A *p* value <0.05 will be considered statistically significant.

**Discussions:**

It is expected that the results of this study will enable the training performed with PThC to be used by health professionals as a new tool to evaluate and prescribe IMT.

**Trial registration:**

ClinicalTrials.gov, NCT02984189. Registered on 6 December 2016.

**Electronic supplementary material:**

The online version of this article (10.1186/s13063-019-3353-0) contains supplementary material, which is available to authorized users.

## Background

Positive results obtained using inspiratory muscle training (IMT) in active and non-active healthy individuals have transformed this type of intervention into one of the most studied in recent decades [1–3]. IMT, which is already used as an integral part of cardiorespiratory rehabilitation in individuals with respiratory muscle weakness [4–6], is currently an alternative for improving the athletic performance of amateur and professional athletes by reducing the sensation of dyspnea in these individuals [7–15].

However, despite the large number of studies that evaluate the efficiency of IMT, there is a gap in the literature related to choosing the best protocol for each population and training objective. Thus, new studies need to be carried out aimed at establishing the best load (intensity), duration and frequency of training for healthy active individuals and to promote central and peripheral adaptations, which can help regulate respiratory muscle activity [16].

Among these parameters, the most studied is the training load, but there is still no consensus as to which is the best load to use. Thus, respiratory muscle strength (RMS) measurements have mainly been used in published studies and the percentage of maximal inspiratory pressure (MIP) is most commonly used for establishing training loads [16, 17]. However, as well as RMS, respiratory muscle endurance (RME) should also be considered, as together they make up the main characteristics of the respiratory system, and can bring about greater systemic adjustments, leading to an improvement in physical performance as the respiratory muscles stand out from the other muscles due to their great resistance [18].

According to Hajghanbari et al. [3], the loads presented in the studies cited in this review used training intensities between 50% and 80% of MIP. In a review by Janssens et al. [19], the authors concluded that changes in inspiratory muscle strength (IMS) were more significant in training with loads between 60% and 80% of MIP. Thus, most of the studies that show good results after the IMT [3, 19] used the 60% load of MIP. However, to the best of our knowledge, no new methods have been published that use the IMS and inspiratory muscle endurance (IME) together to determine the training load.

Based on this, it was proposed in our group’s previous research to determine the critical inspiratory pressure (PThC) of apparently healthy young and middle-aged men during IME [20]. This new methodology to determine the training load was based on the concept of critical power defined as the limit work (W_LIM_) where an exercise can be maintained for a long period of time (T_LIM_) without any signs of fatigue [21, 22] or as the intensity where the aerobic and anaerobic pathways are recruited together [23].

The determination of PThC is performed by the total work (abscissa) graph for the total time to exhaustion (coordinate), from multiple intensities of exercise to fatigue. Once this is done, a linear regression of the points is performed to identify the curvature constant (W′) and the PThC is determined as the point at which the line intersects the abscissa. This work-time relationship is defined by a hyperbolic function, in which the slope represents the aerobic function and the intercept in y (W′), the anaerobic working capacity, that is, the maximal workload that can be performed using stored energy in the active muscle (adenosine triphosphate, phosphocreatine, glycogen and myoglobin-bound oxygen) [24, 25]. In addition, it should be noted that both IMS and IME are used to determine it; the latter by evaluating PTh_MÁX_, which represents the maximal workload that the individual can sustain for at least 1 min [26–29].

Thus, it was observed that the percentage of PThC is similar to other values of critical power identified in the literature, where PThC and the percentage of PTh_MÁX_ are influenced by age [20]. This suggests that the concept of PThC can be applied as a new IMT tool, as this method considers the characteristics of the respiratory musculature, i.e., IMS and IME together [20].

In addition, during the proposition of a new training load, the results of this new methodology need to be compared with the other loads found in the literature to clarify what the best training intensity is for a given population and to define the responses generated by this training in the main systems involved: the cardiovascular, respiratory and metabolic systems.

IMT using PThC can bring additional benefits, such as increased oxygen uptake (VO_2PEAK_), generated by using a moderate to high intensity load that can be performed over a long period of time [23] due to improving the efficiency of respiratory, cardiovascular and musculoskeletal systems and which will contribute to improved physical performance. In addition, a longer delayed mechanism of inspiratory muscle metaboreflex has been pointed out as one of the main factors responsible for the benefits of IMT in healthy individuals [3, 19].

## Methods

### Aim

The aim of this study is to propose a new methodology to determine the IMT load for recreational cyclists, which promotes better results for physical performance and for cardiovascular, respiratory and metabolic responses compared to traditional methodologies.

#### Primary outcome measures

The primary outcome measure is the performance on exercise: peak oxygen uptake (VO_2PEAK_) and work load (W) were obtained by cardiopulmonary exercise testing (CPET).

#### Secondary outcome measures

The secondary outcome measures are as follows:

- The cardiovascular responses to IMT: systolic arterial pressure (SAP), dyastolic arterial pressure (DAP), heart rate (HR), cardiac output (CO), stroke volume (SV) and total peripheral vascular resistance (PVR) will be evaluated during the CPET and incremental inspiratory muscle endurance test (iIME).
- The respiratory responses to IMT: carbon dioxide production (VCO_2_), respiratory exchange rate (RER), respiratory rate (RR), respiratory ventilation (VE), oxygen uptake efficiency slope (OUES) and minute ventilation-carbon dioxide production slope (VE/VCO_2SLOPE_) will be monitored and registered during CPET and iIME. MIP and MEP will be collected in the RMS test. The pulmonary function test (PFT) index comprises: slow and forced vital capacity (SVC and FVC), maximal voluntary ventilation (MVV), forced expiratory volume in 1 s (FEV_1_), relationship between FEV_1_ and CVF (FEV_1_/FVC), IC, expiratory reserve volume (ERV) and MVV.
- The metabolic responses to IMT: oxyhemoglobin (O_2_Hb), deoxyhemoglobin (HHb) and total hemoglobin (tHb) and VO_2PEAK_ responses will be measured during CPET and iIME.

### Study design

This is an experimental, longitudinal, randomized, controlled and double-blind study. The research participants and the researcher (PRS), who will perform the evaluation and reevaluation protocols and statistical analysis, will be blinded. The researcher who will carry out the study analyses (tabulation, data processing and statistical analyses) will not participate in the randomization and training of the participants. Thus, after tabulating all the data, the same person (PRS) will receive a table of data from the other researchers, separated by training groups, using codes to perform the statistical analysis. The participants will be blinded to the training loads to which they are being submitted, by means of a barrier fixed on the computer screen. The methodological design was based on the Standard Protocol Items: Recommendations for Interventional Trials (SPIRIT) (Additional file 1) [30].

### Subjects

Thirty recreational male cyclists, aged between 20 and 40 years, will be evaluated. They will be randomly divided into three groups: sham group (SG), PThC group (CPG) and 60% of the MIP group (60G). The groups will be formed by the randomization of the paired individuals with the same aerobic functional classification [31] and age group (subdivided into decades), using R software 3.0.2 to allocate the subjects.

The criteria used to select the participants will be as follows: male subjects aged 20 to 40 years old, apparently healthy, who have been practicing cycling for at least 6 consecutive months and at least 150 min/week (classified as active by the American College of Sports Medicine [32]). In addition, participants must not have abnormalities in the cardiovascular, respiratory, musculoskeletal or neurological systems, nor of other systems that make the proposed tests impossible. The exclusion criteria of the study are as follows: participants with electrocardiogram (ECG) changes (ischemia, overload, severe arrhythmias (such as ventricular tachycardia) and conduction disorders), both at rest and during the clinical exercise test, obesity (body mass index (BMI) >30 kg/m^2^), diabetes mellitus, hypertension, alterations in the results of laboratory tests, participants with respiratory muscle weakness (MIP<60% predicted [33]), smokers or former smokers with at least 1 year of interruption, alcoholics and users of illicit drugs or medication that may interfere with the results of the research and participants who have performed some type of IMT the last 12 months.

The participants will be recruited by electronic and printed media and by contact with subjects included in the database of the Laboratory of Cardiovascular Physiotherapy (LFCV) at the Federal University of São Carlos (UFSCar). Once identified, eligible participants will be invited to participate in the study and after they have accepted, they will carry out all the evaluations described subsequently and the type of training to which they are allocated.

### Study planning

The present study will be conducted in accordance with the Helsinki Declaration. This study was approved by the Human Research Ethics Committee at the Federal University of São Carlos (UFSCar) (number 1.558.731). All subjects will provide signed informed consent.

The experimental tests and procedures will be performed at the LFCV at the Nucleus of Research in Physical Exercise (NUPEF) at the Department of Physical Therapy (DFisio) and the clinical ergometric tests at the Cardiovascular Physiotherapy area of the School Health Unit (USE), both located at UFSCar, São Carlos campus. The blood tests will be performed at the Clinical Analysis Laboratory at UNIMED (a cooperative medical system) in São Carlos (UNILAB).

Environmental conditions will be controlled, and participants will be instructed according to Perseguini et al. [34]. The tests will always be performed in the afternoon, considering the influence of the circadian cycle on the evaluation results.

On the day of the tests, previously to applying the experimental protocols, the conditions related to the participants’ state of health will be observed. Moreover, prior to the tests, participants will be familiarized with the equipment, respiratory maneuvers and the subjective perception of effort scale – BORG/CR10 [35] in order to reduce participants’ anxiety and prevent the effect of the learning process from affecting the search results.

### Clinical evaluation and characterization of the sample

Before performing the experimental protocols, the participants will be submitted to the following evaluations: anamnesis, conventional 12-lead ECG and treadmill clinical exercise test, performed in the presence of a cardiologist, assisted by a physiotherapist. The aim of these tests will be the clinical and cardiovascular evaluation of the participants.

To characterize the participants and verify their health status, the following evaluations will be performed before the experimental protocol: body composition evaluation by dual-energy x-ray absorptiometry (DXA) (Discovery DXA System, Hologic, USA), nutritional assessment and blood tests (lipid profile, total cholesterol, high density lipoproteins (HDL), low density lipoproteins (LDL), urea, creatinine, fasting glucose, uric acid, insulin resistance, C-reative protein and glycated hemoglobin, to check their health status.

### Experimental protocol

**Fig. 1:**
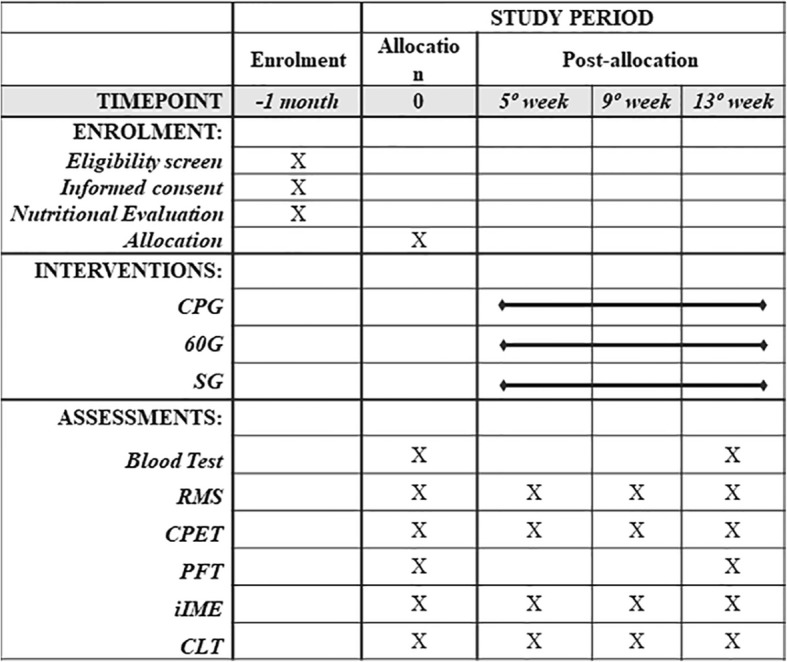
Template of recommended interventional trials (SPIRIT) schedule. 60G, 60% of maximal inspiratory pressure group; CLT, constant load test; CPET, cardiopulmonary exercise test; iIME, incremental inspiratory muscle endurance test; CPG, critical inspiratory pressure group; PFT, pulmonary function test; RMS, respiratory muscle strength test; SG, sham group; sham, 6 cmH_2_O

The experimental protocol will take place over 13 weeks, as shown in Fig. 1. In the 1st, 5th, 9th and 13th weeks, the participants will be submitted to the following evaluations: CPET, RMS, iIME and constant load test (CLT). The PFT will be performed only in the 1st and 13th weeks of the study. The IMT will be carried out for 11 weeks. It is emphasized that the participants will continue doing the training during the reevaluation weeks. The aim of the reevaluations, which will be carried out in the 5th and 9th weeks of training, is to readjust the training loads and to follow the responses to the IMT.

### Respiratory muscle strength tests (RMS)

The evaluation of RMS will be performed with the participants at rest in the sitting position, using a digital manovacuometer (MVD-300, Globalmed, Porto Alegre, Brazil) and a nasal clip, according to the Brazilian guidelines for measuring maximal static respiratory pressures [36]. This measure will always be carried out by the same appraiser.

MIP will be determined after maximal inspiratory effort, from the residual volume. The MEP will be determined after maximal expiratory effort, from total lung capacity. These maneuvers will be performed against a rigid tube, occluded distally and a 2-mm-hole mouthpiece [36] will be used. The values of the maximal respiratory pressures will be those observed in the first second after the peak of pressure [36]. At least three maneuvers will be performed, with a 30-s interval between each maneuver [37], obtaining the highest reproducible values (difference < 10%) found in at least three maneuvers and considering the maximal respiratory pressure as the highest value. Normal values will be based on the regression equation proposed by Neder et al. [38] for the Brazilian population. Respiratory muscle weakness, maximal static pressure values < 60% of those predicted, will be considered [33].

### Cardiopulmonary exercise test (CPET)

The CPET will be used to assess the aerobic power of the participants’ VO_2 PEAK_ [39] to determine the gas exchange threshold (GET) by the ventilatory method [40], the respiratory compensation point (RCP) [39] and inspiratory muscle metaboreflex [41].

A ramp-type protocol will be performed on an electromagnetic braking cycle ergometer (Corival V3, Lode BV, The Netherlands) and will consist of a 6-min rest, 3-min free-load warm up (0 watts) and a gradual increase in load until the exercise is stopped, followed by 6 min active recovery (25 watts) and 1 min passive (without pedaling) recovery. The power increment will be calculated for each participant according to the values established by the formula described by Wasserman et al. [42] and adapted according to the evaluator’s experience, preventing the increment from being underestimated.

Participants will be instructed to maintain a cadence between 60 and 80 rpm throughout the protocol and the test will last from 8 to 12 min [42]. Three independent evaluators will determine the GET and the RCP. The highest value of VO_2_ obtained in the last 30 s of the CPET will be considered the VO_2PEAK_ [39]. In addition, the following variables will be evaluated in the GET and peak effort: VO_2_, VCO_2_, VE, OUES and VE/VCO_2_ slope [39, 43].

The activation of the inspiratory muscle metaboreflex will be evaluated by analyzing the patterns of the variables: O_2_Hb, HHb and tHb obtained by near infrared spectroscopy (NIRS) and the cardiovascular data, HR and mean arterial pressure (MAP), obtained by a bioamplifier for ECG signals (BioAmp FE132) and a Finometer (Finapres Medical Systems, The Netherlands), respectively, at the intensities of 50–100% of the VO_2PEAK_, subdivided into 10% intervals. In addition, the activation point of the inspiratory muscle metaboreflex will be considered the moment at which the nonlinear HR, MAP and oxygenation decrease for the vastus lateralis (VL) and increase for the external intercostal (EI), i.e., redirection of blood flow from the peripheral musculature to the respiratory muscles [6].

### Pulmonary function test (PFT)

This examination will be performed according to the international standard [44] and will consist of tests of SVC, FVC and maximal MVV. The test will be performed using a flow module coupled with a system of ventilatory and metabolic measurements (Ultima MedGraphics - St. Paul, MN, USA). The variables to be analyzed are SVC, FVC, FEV_1_, FEV_1_/FVC, IC, ERV and MVV. The predicted values will be calculated according to Pereira [45].

### Protocol for the determination of critical inspiratory pressure (PThC)

**Fig. 2:**
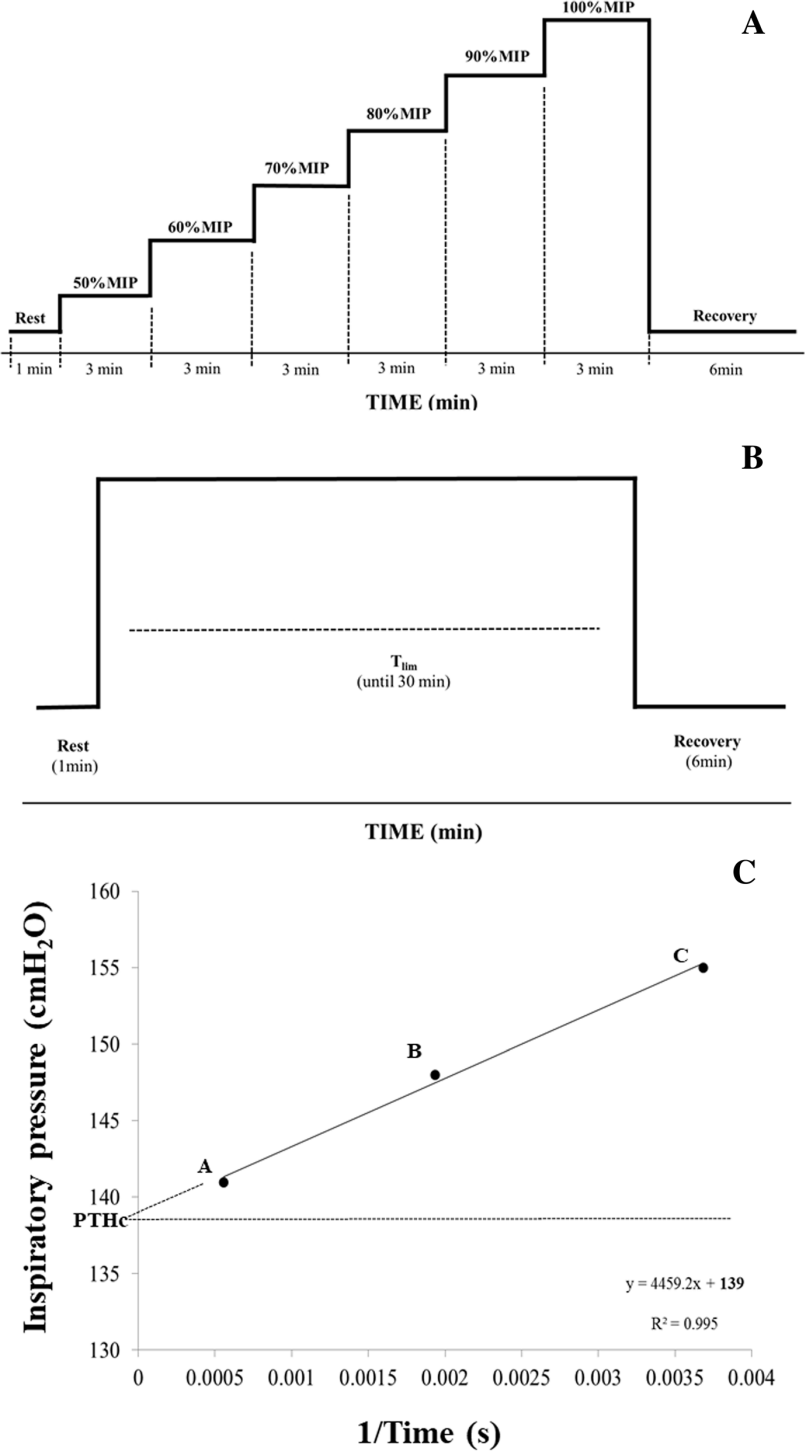
Description of steps to calculate critical inspiratory pressure. **a** Incremental inspiratory muscle endurance test. **b** Constant load test. **c** Linear regression to determination of critical inspiratory pressure (PThC). MIP, maximal inspiratory pressure; min, minute; REC, recovery

An incremental protocol with a load of 50–100% MIP (Fig. 2) will be performed and 10% of MIP will be added every 3 min. The participant will receive a verbal stimulus to maintain the RR at 12 breaths/minute and the test may continue until he reaches 100% of MIP. In this case, if the participant can generate an air flow capable of triggering the equipment more than once at this load, the MIP measurement and the test will be repeated. The following will be considered: failure to maintain the stipulated load at 12 breaths/minute for at least 1 min, failure to maintain respiratory effort indicated by the participant (BORG/CR10 ≥ 7) [35] and the participant requesting interruption. The highest percentage of MIP that the participant is able to maintain for at least 1 min (PTh_MÁX_) will be stipulated as the IME measurement [26–28, 46].

After determining the PTh_MÁX_, individuals will carry out incursions against a constant load, without the RR being controlled, in order to identify the total time tolerated in each load (T_LIM_). Individuals will be exposed to three different resistances (95%, 100% and 105% PTh_MÁX_) with a 15-min interval between them. The order of resistances will be determined by holding a draw without the individual knowing the order of the charges in which he will be subjected to.

After obtaining the T_LIM_ of each load, a pressure graph will be plotted by time and the PThC will be determined by linear regression between the variables [23].

### Monitoring the experimental protocol

The equipment described subsequently will be used to monitor the participants in the evaluations of the CPET and iIME.

### Metabolic and respiratory variables

The ventilatory and metabolic variables will be collected, breath by breath, through a system of expired gas measurements (Ultima MedGraphics - St. Paul, MN, USA) and processed using specific software (Breeze Suite 7.1, MedGraphics - St. Paul, MN, USA). In addition, the BORG/CR10 scale [35] will be used to assess the participant’s subjective perception of the exercise.

### Cardiovascular variables

The acquisition of the ECG and AP signals to evaluate the cardiovascular responses will be performed at a sampling frequency of 1.000 Hz. The ECG signals will be captured by means of the CM5 lead. The HR will be recorded and stored beat-to-beat. Electrocardiographic signals will be captured and processed via an interface between a bioamplifier for ECG signals (BioAmp FE132, ADInstruments, Australia) and a biological signal acquisition system (Power Lab 8/35, ADInstruments, Australia) and a microcomputer (Intel I5).

On the other hand, pulse pressure will be captured using Finometer Pro® (Finapres Medical Systems, The Netherlands), which allows non-invasive measurements of pulse arterial pressure (FinAP), beat-to-beat, obtained by positioning a cuff on the third phalanx of the third finger of the left hand. The equipment will be calibrated according to the manufacturer’s instructions. In addition to pulse AP, the values of CO, SV and PVR, derived from the AP curves and analyzed using Beat Scope® Easy software (Finapress Medical Systems, The Netherlands) will be evaluated.

### Metabolic evaluation by NIRS

The EI and VL muscle oxygenation variables will be measured by the NIRS (Oxymon Mk III, Artinis Medical Systems, The Netherlands). Two optodes will be used, which will be positioned as described below subsequently: IE = eighth left intercostal space in the anterior axillary line; VL = 12–15 cm of the knee joint, lateral line between the greater trochanter of the femur and the patella [47].

The sampling rate of the device will be set at 250 Hz. Inter-ops distance will be 35 mm for the EI and 40 for the VL [47]. The differential path-length factor (DPF) will be 4 for the EI muscles and 3.83 for the VL. The device will be recalibrated for each subject and the data will be continuously captured during the CPET and the iIME. For each muscle, the change in tissue oxygenation and local blood volume will be estimated by changes in O_2_Hb, HHb and tHb, calculated automatically by the equipment software using the formula (tHB = O_2_Hb + HHb).

### Inspiratory muscle training (IMT)

#### Training description

**Fig. 3:**
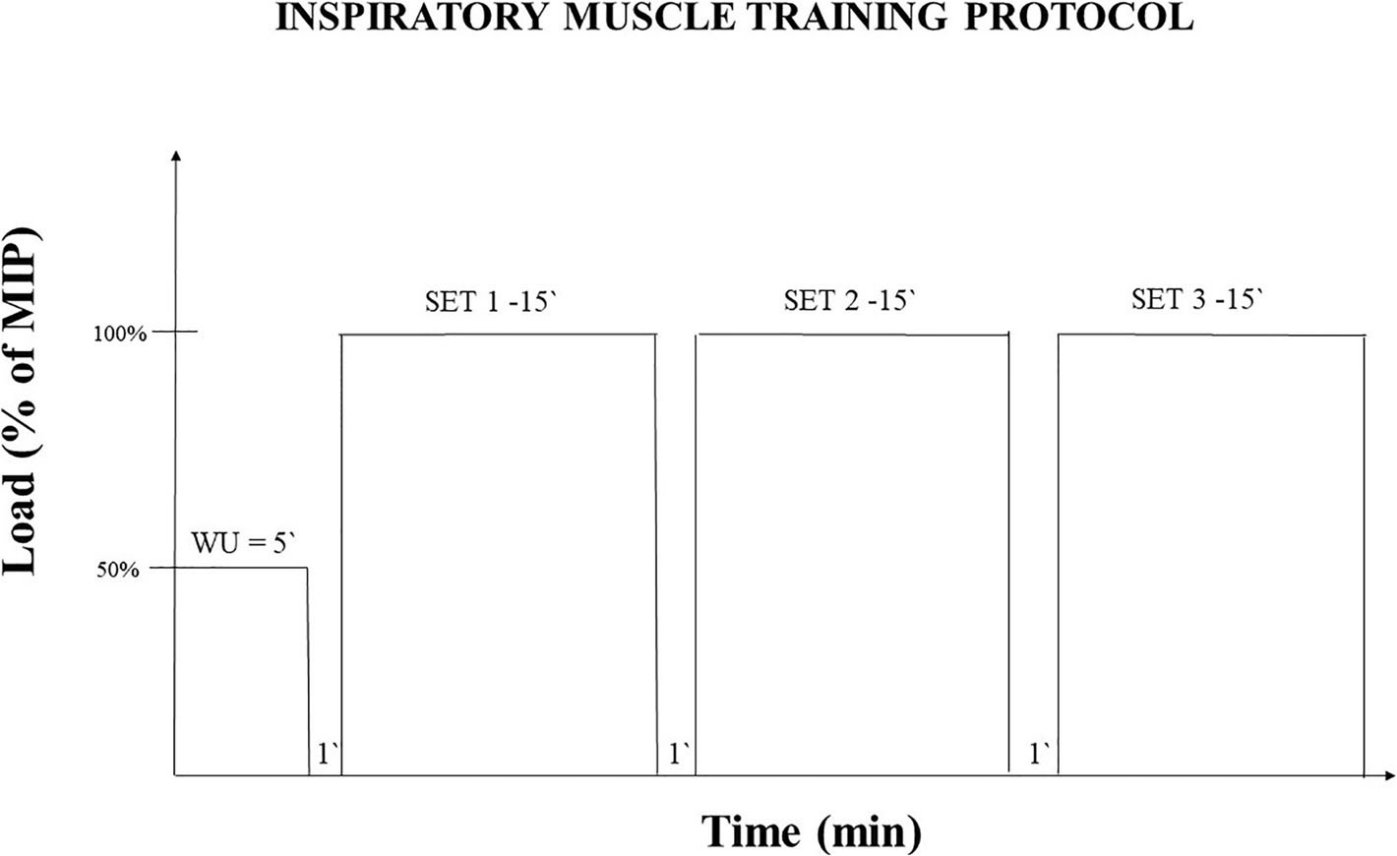
Schematization of inspiratory muscle training protocol. MIP, maximal inspiratory pressure; min, minute; WU, warm-up

The training will last for 11 weeks, with a weekly frequency of three sessions and each session will last 1 h. Each session will consist of a 5-min warm up, where each volunteer will perform a constant loading protocol with 50% of their training load. The training protocol will consist of three sets of 15 min of breaths, with a 1-min interval between them (Fig. 3).

The loads that will be used in the training will be as follows: for the CPG the value of the PThC, for the 60G, the resistance will be 60% of MIP and for the SG, the training will be carried out with a resistance of 6 cmH_2_O. All groups will perform IMT using the linear load respiratory (PowerBreathe inspiratory muscle trainer, Ironman K5, HaB Ltd., UK).

The participants will be instructed to maintain diaphragmatic breathing and RR of 12 breaths/minute for the entire duration of the training protocol, and the RR will be controlled using a recorded verbal command, ensuring that all participants receive the same stimulus. During the 11 weeks of training, participants will be instructed not to change their activities and physical training or food intake.

Every day, before and after the training protocol, the AP will be checked, and the health status of the participants will be observed while the HR is monitored, recorded and stored during all training sessions using a Polar 800CX (Kempele, Finland).

In addition, during the training period, the participants will be asked to complete a physical activity schedule to monitor the activities performed by each individual during the research participation and complete a food survey that will be analyzed later by a nutritionist, thus avoiding these factors interfering with training responses. The weekly training volume and the end, will be controlled over 11 weeks and ensured for all participants throughout this study.

Participants who do not complete the three weekly training sessions and/or the total of 33 training sessions will be excluded during the survey, as will participants who change their physical activities or their eating habits or those who begin to use any supplement or medication continuously.

#### Statistical analysis

Due to the lack of studies that evaluate the effect of different loads of IMT, among them PThC, on the physical performance in recreational cyclists, we chose to carry out the statistical analysis considering the desired study effect size as proposed by Cohen [48]. Thus, the sample calculation was performed using GPower software 3.1.3 to determine the sample size, the mean effect size (*f* = 0.25, according to Cohen [48]) was used for the two-way mixed analysis of variance (ANOVA) and values of 0.05 for the type I probability error (α) and 0.80 for the type II probability error (power or β). Therefore, in order to ensure these pre-established conditions, at least 30 subjects should participate in this study.

All statistical analyses will be processed using the SPSS Statistics software 17.0 (SPSS Inc., Chicago, IL, USA). The level of significance will be set at *p* < 0.05. Normally distributed data will be presented according to the mean ± standard deviation and non-normally distributed data according to the median (interquartile range).

The Shapiro-Wilk test will be used to test for normal distribution and the Levene test for evaluation of homogeneity. A descriptive analysis of the three groups evaluated and then the paired *t* test will be performed on the following data: age, anthropometric evaluation, body composition densitometric analysis and blood test results.

The three groups will be compared considering the factors of group and training time, in terms of the variables PFT (FVC, FEV_1_/FVC, IC, ERV and MVV), RMS (MIP and MEP), CPET (VO_2PEAK_, VE, RR, OUES and VE/VCO_2_slope), cardiovascular variables (HR, CO, SAP, SV, total PVR) and metabolic variables (O_2_Hb, HHb, tHb and VO_2PEAK_), using two-way mixed ANOVA for parametric data analysis and for non-parametric data analsysis, the data will be mathematically transformed in order to normalize the data.

### Expected results

Among the scientific contributions from this project, the main one refers to adopting a new evaluation methodology (PThC) and prescription of IMT. It is expected that CPG will obtain better results than 60G and SG, such as obtaining a greater increase in workload (Watts) and peak oxygen uptake (VO_2PEAK_) in the CPET; higher MIP and iIME; decreased sensation of dyspnea and the sensation of peripheral fatigue, evaluated by the perception index to the physical effort of BORG/CR10 and delay in the activation of the inspiratory muscle metaboreflex both during the CPET and in the iIME.

Thus, it is expected that the use of IMT based on this new approach can increase the benefits derived from traditional methodology (IMT based on 60% of MIP); that it can provide subsidies on the best understanding of the physiological responses from its application; and that this new methodological approach can be used by health professionals as a new tool to evaluate and prescribe IMT, bringing more satisfactory results and greater physiological impact.

## Discussion

Most systematic reviews [1, 3, 19, 49] that study the effects of IMT emphasize the need for studies with a controlled and randomized experimental design, following the guidelines of the Consolidated Standards of Reporting Trials (CONSORT) [50], and to establish training parameters that seek to achieve the best results for the population studied.

However, adapting this type of training to meet these guidelines becomes extremely complicated because of the need for this therapy to be used in a laboratory. Several experiments, also presented in these reviews [1, 3, 19], perform the training on average five times a day, or often with more than one session on the same day. Therefore, for the results of this research to be discussed considering the studies already published in the literature, we used the determination of the average training volume [51] of these studies, because this is an important parameter for determining training effectiveness, mainly resistance. Moreover, we divided the total time into three training days per week, the number of days also indicated by the American College of Sports Medicine [32], as the ideal time for performing physical activities, thus totaling 55 min of IMT on each day of training.

### Contributions to the area

This study will be important for physiotherapists, physical educators and other health professionals who work with physical exercise and training, as knowledge concerning the cardiovascular, respiratory and metabolic responses generated by IMT in recreational cyclists, using PThC and 60% of MIP, will enable these professionals to prescribe an IMT in a more suitable way for healthy active individuals. It will also help to encourage new studies that aim to promote greater gains in this population.

It is hoped that the results of this study will enable training performed with PThC to be used by health professionals as a new tool for assessing and prescribing IMT.

## Trial status

Status: participant recruitment is currently underway.

Recruitment began in May 2017.

Final recruitment will be December 2019.

## Additional file

Additional file 1: SPIRIT 2013 checklist: Recommended items to address in a clinical trial protocol and related documents. (PDF 112 kb)

## Abbreviations

60G: 60% of maximal inspiratory pressure group
AP: Arterial pressure
BMI: Body mass index
BORG/CR10: Subjective perception of effort scale
CLT: Constant load test
CO: Cardiac output
CONSORT: Consolidated Standards of Reporting Trials
CPET: Cardiopulmonary muscle test
CPG: Critical inspiratory pressure group
DFisio: Department of Physical Therapy
DPF: Differential path-length
DXA: Dual-energy x-ray absorptiometry
ECG: Electrocardiogram
EI: External intercostal
ERV: Expiratory reserve volume
FEV_1_: Forced expiratory volume in 1 s
FEV_1_/FVC: Relationship between FEV_1_ and FVC
FVC: Forced vital capacity
GET: Gas exchange threshold
HDL: High density lipoproteins
HHb: Deoxyhemoglobin
HR: Heart rate
IC: Inspiratory capacity
IME: Inspiratory muscle endurance
IMS: Inspiratory muscle strength
IMT: Inspiratory muscle training
iIME: Incremental inspiratory muscle endurance test
LDL: Low density lipoproteins
LFCV: Laboratory of Cardiovascular Physiotherapy
MAP: Mean arterial pressure
MEP: Maximal expiratory pressure
MIP: Maximal inspiratory pressure
MVV: Maximal voluntary ventilation
NIRS: Near infrared spectroscopy
NUPEF: Nucleus of Research in Physical Exercise
O_2_Hb: Oxyhemoglobin
OUES: Oxygen uptake efficiency slope
PFT: Pulmonary function test
PthC: Critical inspiratory pressure
PTh_MAX_: Maximal sustained respiratory pressure for 1 min
PVR: Peripheral vascular resistance
RCP: Respiratory compensation point
RME: Respiratory muscle endurance
RMS: Respiratory muscle strength test
RR: Respiratory rate
SG: Sham group
SPIRIT: Standard Protocol Items: Recommendations for Interventional Trials
SV: Stroke volume
SVC: Slow vital capacity
tHb: Total hemoglobin
T_LIM_: Inspiratory muscle endurance time
UFSCar: Federal University of São Carlos
UNILAB: Laboratory of Clinical Analysis at UNIMED
UNIMED: Cooperative medical system
USE: Health School Unit
VCO_2_: Carbon dioxide production
VE: Pulmonary ventilation
VE/VCO_2_ slope: Minute ventilation-carbon dioxide production slope
VL: Vastus lateralis
VO_2_: Oxygen uptake
VO_2PEAK_: Peak oxygen uptake
W′: Constant of curvature
W_LIM_: Maximal workload
